# Loop-Mediated Isothermal Amplification Assay for Visual Detection of *Salmonella enterica* Serovar Typhimurium in Food Animal Meat Products

**DOI:** 10.3390/foods14101731

**Published:** 2025-05-13

**Authors:** Rance Derrick N. Pavon, Windell L. Rivera

**Affiliations:** Pathogen-Host-Environment Interactions Research Laboratory, Institute of Biology, College of Science, University of the Philippines Diliman, Quezon City 1101, Philippines; rnpavon@up.edu.ph

**Keywords:** calcein, closed tube, loop-mediated isothermal amplification, *Salmonella enterica* serovar Typhimurium, *STM4497* gene

## Abstract

Detection of *Salmonella*, a highly diverse foodborne pathogen, is paramount to ensure safety and protection of the animal industry and its consumers. *Salmonella enterica* serovar Typhimurium is among the most important non-typhoidal serovars causing gastroenteritis worldwide. However, traditional serovar identification is labor- and resource-intensive, while typical molecular tools require expensive reagents and equipment. Hence, this study developed and optimized a calcein-based and closed-tube loop-mediated isothermal amplification (LAMP)-based assay to detect *S.* Typhimurium following enrichment steps compared with an optimized PCR assay. The PCR assay showed 100% specificity in silico confirmed through DNA sequencing. For actual specificity testing, both PCR and LAMP showed 100% specificity to *S.* Typhimurium. For DNA sensitivity, while PCR showed a limit of detection of 22 pg/μL, LAMP showed a 100-fold higher sensitivity at 220 fg/μL. Meanwhile, for pure culture sensitivity, both assays detected at least 4.98 × 10^4^ CFU/mL. Parallel testing of 208 raw meat samples from wet markets in Metro Manila, Philippines, showed corroboration and statistical association of the optimized PCR and LAMP with 89.42% and 90.87% positivity rates for *S.* Typhimurium, respectively. Hence, the developed closed-tube and calcein-based LAMP assay is potentially a powerful yet simple, sensitive, and fast method for *S.* Typhimurium detection.

## 1. Introduction

With more than 2600 serovars, *Salmonella* possesses diverse pathogenicity and antimicrobial resistance potential, which require rigorous and constant surveillance throughout the food animal value chain [1]. These pathogenic foodborne bacteria are divided into two species, namely *enterica* and *bongori*, with the former further divided into six subspecies of which subspecies I or *enterica* contain most serovars that contribute to human and animal salmonellosis [2]. Non-typhoidal *Salmonella* (NTS) cause gastroenteritis, with 93.8 million cases and 155,000 deaths annually worldwide [3]. *Salmonella enterica* subspecies *enterica* serovar Typhimurium is one of the most prevalent and important NTS, affecting multiple hosts, contaminating animal products during production, processing, and distribution, and it is frequently associated with human food poisoning and outbreaks [4,5,6,7]. *S.* Typhimurium infections typically last four to seven days and are often self-limiting; however, younger, older, or immunocompromised individuals are more susceptible to severe, invasive, and disseminated infections that require antibiotic therapy [8]. In the Philippines, *Salmonella* was reported to be the highest microbiological cause of food poisoning outbreaks from 2005 to 2018 [9]. High prevalence of *Salmonella*, determined through conventional PCR methods, has also been reported in abattoirs and wet markets in Metro Manila [10,11]. The most reported NTS serovars in clinical cases in the Philippines are Typhimurium and Enteritidis. Interestingly, these serovars have also been detected in meat samples from wet markets of Metro Manila using serological, molecular, and genomic methods [12,13]. However, these studies mainly implement conventional culture, serological testing, and PCR methods for *Salmonella* detection and serovar identification, which require selection and isolation while underestimating serovar prevalence and other drawbacks.

Detection and identification of *Salmonella* are crucial in preventing outbreaks and economic losses, which mainly rely on standard conventional culture techniques [14] that are time-consuming (about 5–7 days to obtain results), labor- and resource-intensive, and expensive, thus limiting testing capacities. Traditional *Salmonella* serotyping using the phenotypic Kauffman–White–Le Minor (KWL) scheme requires the storage and use of large volumes of >250 antisera and 350 antigens, which are also susceptible to variable phenotypic antigenic expressions [15,16]. Recent molecular detection methods such as PCR and qPCR have increased capacity and lessened turnaround time but remain costly and require technical skills, expertise, and expensive equipment [17]. Meanwhile, Notomi et al. [18] pioneered loop-mediated isothermal amplification (LAMP) detection method, which is simple, cost-efficient, highly specific, and sensitive, with a shorter turnaround time, ideal for surveillance, small-scale, and routine laboratories. This technique has gained traction even for *Salmonella* detection [19], with some studies detecting important serovars such as Typhimurium and Enteritidis with diverse visualization platforms [5,6,20]. In most LAMP assays, multiple cap-opening procedures, or open-tube protocols, are implemented to add polymerases, dyes and perform gel electrophoresis, which are potential cross-contamination events [21,22,23]. Currently, there are numerous LAMP visualization platforms available. Turbidity observations are subjective and often have insufficient contrasts for naked eye differentiation. DNA intercalating dyes, such as SYBR Green I, Eva Green, Midori Green, and Quant-iT PicoGreen, although highly sensitive, are toxic and require open-tube procedures for their addition after amplification, which are highly prone to carry-over contaminations [24]. Meanwhile, metal indicator dyes, such as hydroxynaphthol blue (HNB) and calcein, are safer and applicable for closed-tube procedures [24,25]. Several studies have reported the use of calcein-based LAMP assays for detecting *Salmonella* spp. [26,27,28], including one study specifically targeting the Enteritidis serovar [20]. However, little to no studies have been conducted on calcein-based LAMP for *S.* Typhimurium detection. Meanwhile, closed-tube protocols allow visualization through colorimetry or fluorescence using metal indicator dyes with lower contamination risks [23,29]. Hence, this study aimed to develop a closed-tube, calcein-based LAMP detection assay for the detection of *S.* Typhimurium following enrichment steps and to evaluate its performance in parallel with conventional PCR. The developed method has the potential to be a standard, simple, and fast method for the detection of *S.* Typhimurium in diagnostic food laboratories in the Philippines.

## 2. Materials and Methods

### 2.1. Raw Meat Sample Collection

Unprocessed and raw meat samples from three animal sources, namely chicken, swine, and cow, were purchased from retail wet markets around Metro Manila, Philippines, for processing and analysis. A total of 208 raw meat samples, including chicken (*n* = 81), beef (*n* = 66), and pork (*n* = 61), were collected from retail stalls and stored in a cooler container containing ice (approximately 4–5 °C). They were then transported to the Pathogen-Host-Environment Interactions Research Laboratory (PHEIRL) at the Institute of Biology, University of the Philippines Diliman, for immediate processing and culture for the detection of *Salmonella* spp. through traditional enrichment and conventional PCR assay. After *Salmonella* confirmation, samples were then run through the optimized LAMP assay parallel with the optimized conventional PCR assay for detection of *S. enterica* serovar Typhimurium using the *STM4497* gene.

### 2.2. Conventional Culture of Salmonella in Raw Meat Samples

For the conventional culture method, the protocol followed standard procedures [14]. Each meat sample bag collected from retail stalls was aseptically opened, and 25 g of the sample was minced with flame-sterilized scissors and forceps and placed in sterile stomacher bags (Whirl-Pak^®^, Nasco, WI, USA). A volume of 225 mL of buffered peptone water (BPW) (BD Diagnostics System, NJ, USA) was then added, followed by homogenization using the BagMixer^®^ 400 (Interscience, Saint-Nom-la-Bretèche, France) for 30 s and incubation for 18–24 h at 37 °C as the pre-enrichment step. Then, 100 μL of the resulting BPW culture was transferred to 10 mL Rappaport Vassiliadis (RV) broth (BD Diagnostics System, NJ, USA) and incubated at 42 °C for 18–24 h for selective enrichment. Following this selective enrichment step, 1 mL of the RV broth was collected in sterile microcentrifuge tubes for washing and DNA extraction processes.

### 2.3. DNA Extraction via Boiling Lysis Method

RV cultures were subjected to DNA extraction through the boiling lysis method [11,30]. Briefly, 1 mL of RV cultures from conventional culture methods was centrifuged at 15,330× *g* for 5 min. The cell pellets collected were then subjected to washing, while the supernatants were discarded. Washing of the cell pellets was performed twice, involving 1 mL of 1x phosphate-buffered saline (PBS) solution to resuspend the collected cell pellets followed by re-centrifugation at 15,330× *g* for 5 min. After the second washing step, cell pellets were then resuspended with 1x Tris-EDTA (TE) buffer and were subjected to boiling at 100 °C for 10 min. The resulting TE solutions were then centrifuged at 2656× *g* for 5 min. The supernatants generated after centrifugation containing the DNA were then transferred to a new sterile microcentrifuge tube and stored in a freezer (−20 °C) for downstream processing.

### 2.4. Conventional PCR Assay for Salmonella Detection

Following the protocol of Ng and Rivera [10], DNA extracts from the enrichments were subjected to *invA* gene detection with primers from Chiu and Ou [31] for *Salmonella* confirmation. Each PCR reaction mixture was 12.5 μL in volume, consisting of 1× GoTaq Green Master Mix (Promega Corporation, Fitchburg, WI, USA), 0.4 μM forward and reverse primers for the *invA* gene, 1 μL DNA template, and nuclease-free water to make up the total volume. The PCR assay followed these conditions: initial denaturation at 95 °C for 2 min, then 30 cycles of denaturation at 95 °C for 30 s, annealing at 60 °C for 30 s, and extension at 72 °C for 2 min, with a final extension at 72 °C for 5 min using a thermocycler. *S. enterica* subsp. *enterica* serovar Typhimurium American Type Culture Collection (ATCC) strain 14028 was used as the positive control, while *Escherichia coli* ATCC strain 25922 was used as the negative control, and nuclease-free water was used as the no-template control. The primer sequences and references for the *invA* gene are found in Table 1.

### 2.5. Optimization of PCR Assay for Salmonella Serovar Typhimurium Detection

In silico PCR amplification was first performed for initial verification of the outer primers F3 and B3 targeting the *STM4497* gene [32] using in silico PCR through http://insilico.ehu.eus/PCR/ (accessed on 3 February 2025) [33] against 422 up-to-date bacterial genome sequences from NCBI to determine and verify amplicon product sizes and primer specificity to *S.* Typhimurium. This web-based tool calculates theoretical PCR results after specifying the primer sequences, maximum length of amplicons, and bacterial genera to be used. The PCR assay for the detection of *S.* Typhimurium was optimized under gradient PCR for the annealing step temperature of F3 and B3 outer primers targeting the *STM4497* gene in triplicate and repeated three times for replicability and reproducibility. Each PCR reaction consisted of GoTaq^®^ G2 Master Mix (Promega Corporation, WI, USA), F3 and B3 primers [32], nuclease-free water, and DNA template, following standard concentrations. Similar PCR conditions to *invA* gene detection were implemented except for annealing temperature and performed using a thermocycler. *S.* Typhimurium ATCC strain 14028 was used as the positive control, while *S.* Enteritidis strain 13076 was used as the negative control, and nuclease-free water was used as the no-template control. DNA sequencing through Macrogen (Seoul, Republic of Korea), sequence analysis through Mega version 11 [34], and NCBI basic local alignment search tool (BLAST) of four PCR products (one from the ATCC control and three from *STM4497*-positive raw meat samples) were used to confirm product identity. The primer sequences and references for *STM4497* F3 and B3 primers can be found in Table 1.

### 2.6. Optimization of LAMP Assay for S. Typhimurium Detection

LAMP reactions were based on several protocols [19,32,35,36] with some modifications and were run in triplicate and repeated three times for replicability and reproducibility. The closed-tube, calcein-based LAMP components and concentrations used in this study were previously optimized [36], except for the betaine concentration, which was specifically optimized in the current study. Each LAMP reaction mixture was 25 μL in volume and consisted of 1x isothermal amplification buffer (New England Biolabs, Inc., Ipswich, MA, USA), a premixed reaction buffer involving Tris-HCl, (NH_4_)_2_SO_4_, KCl, MgSO_4_, Tween 20, and MnCl_2_. Calcein-based fluorescence was implemented for this study, with 64 μM of calcein, 3 μM of added MgSO_4_, and 1 μM of added MnCl_2_ [36]. dNTPs (A, C, G, T nucleotides) at 1.4 mM each were mixed (Vivantis, Subang Jaya, Malaysia) and added to serve as building blocks for DNA replication at various concentrations. The LAMP primers for *S. enterica* subsp. *enterica* serovar Typhimurium detection were based on Azinheiro et al. [32], targeting the *STM4497* gene with five primers (FIP, BIP, F3, B3, and LoopF). The primer sequences and references targeting the *STM4497* gene can be found in Table 1. Betaine (Sigma-Aldrich, Inc., St. Louis, MO, USA), which enhances nucleic acid amplification by reducing secondary structure formation in GC-rich DNA and increasing reaction specificity [6,37], was also included in the assay. Its concentration was optimized using four levels: 1.0 M, 1.1 M, 1.4 M, and 1.5 M. Then, nuclease-free water was also added to make up the total volume of 25 μL. Lastly, 320 U/mL of *Bst* DNA polymerase enzyme 2.0 (New England Biolabs, Inc., MA, USA) was added to each reaction separately. Then, 1 μL of DNA template was added prior to closing of tubes for LAMP incubation at 65 °C for 60 min, followed by deactivation at 80 °C for 2 min, using a thermocycler. *S.* Typhimurium ATCC strain 14028 was used as the positive control, while *S.* Enteritidis strain 13076 was used as the negative control, and nuclease-free water was used as the no-template control. Tubes with yellow-green color ascertained by the naked eye or fluorescence under a blue light LED/UV transilluminator were considered positive for *S.* Typhimurium, while tubes with orange-brown color and absence of fluorescence were considered negative. For verification, LAMP amplicons were also run through gel electrophoresis, with ladder-like bands indicating a positive result. *S.* Typhimurium ATCC 14028 served as the positive control.

### 2.7. Specificity and Sensitivity of PCR and LAMP Assays for S. Typhimurium Detection

Nine (*n* = 9) ATCC *Salmonella* serovar strains (Anatum 9270, Choleraesuis 7001, Choleraesuis 10708, Enteritidis 49223, Enteritidis 13076, Heidelberg 8326, Newport 6962, Typhimurium 14028, and Typhimurium 25241) and nine (*n* = 9) non-*Salmonella* strains (*Acinetobacter baumanii* BAA 1605, *Escherichia coli* 25922, *Escherichia coli* O157:H7 43888, *Escherichia coli* O78:H11 35401, *Enterococcus faecalis* 14506, *Klebsiella pneumoniae* 13883, *Klebsiella pneumoniae* 700603, *Pseudomonas aeruginosa* 10145, and *Vibrio parahaemolyticus* 13204) in glycerol stocks were subjected to a revival process [38] prior to specificity testing. Briefly, 100 μL of glycerol stocks was transferred to fresh TSB and incubated at 37 °C for 18–24 h. Cultures were then subjected to the DNA extraction process via the boiling lysis method. The DNA extracts collected were then subjected to both the optimized PCR and LAMP assays for *STM4497* gene detection for specificity testing in triplicate and repeated three times for replicability and reproducibility, with nuclease-free water serving as the no-template control.

To determine and compare the sensitivity of PCR and LAMP assays, various protocols were followed with some modifications [5,6,20,21,32]. For DNA dilution sensitivity, three to four colonies from an overnight (18–24 h) and TSA culture of *S.* Typhimurium ATCC 14028 were resuspended in 1x TE buffer and subjected to DNA extraction via boiling lysis followed by a 10-fold serial dilution using 1x TE buffer. Then, the suspensions were subjected to both the optimized PCR and LAMP assays for *STM4497* (*S.* Typhimurium) gene detection for sensitivity testing in triplicate and repeated three times for replicability and reproducibility, with nuclease-free water serving as the no-template control. DNA concentrations were read under Multiskan SkyHigh Microplate Spectrophotometer (Thermo Fisher Scientific, Inc., Waltham, MA, USA) with a μDrop™ Duo plate with 1X TE buffer as the reagent blank and run in duplicate to determine the limit of detection (LOD) in ng/μL. Concentrations less than 1 ng/μL were calculated from the concentration of the stock DNA extract due to the LOD of the spectrophotometer to prevent inaccurate readings. For pure bacterial culture sensitivity, an overnight TSB culture of *S.* Typhimurium 14028 was standardized to 0.5 McFarland and was subjected to a 10-fold serial dilution using nuclease-free water in which 10 μL was spread-plated in TSA and incubated at 37 °C to measure CFU/mL. The solutions were also subjected to a DNA extraction process using the boiling lysis method in 1x TE buffer and were run in both the optimized PCR and LAMP assays for *STM4497* gene detection in triplicate and repeated three times for replicability and reproducibility, with nuclease-free water serving as the no-template control.

### 2.8. Detection of S. Typhimurium from Raw Meat Samples

For the evaluation and validation of the optimized closed-tube calcein-based LAMP assay for *S.* Typhimurium detection, parallel testing of the optimized PCR and LAMP assays was performed on DNA extracts from conventional culture enrichments. A total of 208 DNA extracts representing 208 raw meat samples collected from retail wet markets were run in both PCR and LAMP assays, followed by data analysis for method comparison.

### 2.9. Gel Electrophoresis and Visualization

Amplicons in both PCR and LAMP assays were run in gel electrophoresis using 2% agarose gels (Vivantis, Subang Jaya, Malaysia) with 1x Tris-acetate-EDTA (TAE) buffer using 10,000× GelRed^®^ nucleic acid gel stain (Biotium, Fremont, CA, USA). Four microliters of PCR products was then loaded in each well, with a 100 bp molecular weight ladder (Bioline, Essex, UK) as the weight marker. Electrophoresis was conducted at 280 V for 45 min using the CBS Scientific gel electrophoresis system (Thermo Fisher Scientific, MA, USA) containing 1x TAE solution as the running buffer. Gels were then viewed under ultraviolet light using the Vilber Lourmat gel documentation system (Vilber, Marne-la-Vallée, France) at 265 nm.

### 2.10. Data Analysis

The performance of the developed and optimized LAMP assay for *STM4497* gene detection was determined and validated via comparison with the optimized PCR method, which served as the reference assay. Descriptive statistical analysis using Fisher’s exact test was performed in SPSS version 26 software (IBM, Armonk, NY, USA) to determine significant associations between the two methods in actual sample testing [39] to evaluate the potential of the optimized LAMP assay as a suitable *S.* Typhimurium detection tool.

## 3. Results

### 3.1. Optimized PCR and LAMP Detection of S. Typhimurium Using the STM4497 Gene

**Figure 1:**
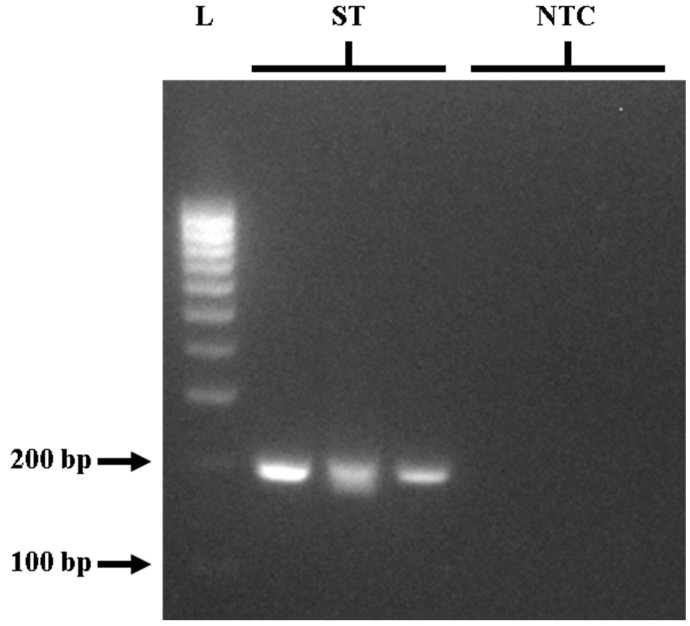
Gel electrophoresis of PCR targeting the *STM4497* gene in triplicate showing around 213 bp product. L—100 bp DNA molecular weight ladder; ST—*S.* Typhimurium; NTC—no-template control.

To provide a comparison of PCR and LAMP assays for *STM4497* gene detection, the outer primers (F3 and B3) from Azinheiro et al. [32] were initially optimized for PCR detection. Following the *invA* gene PCR protocol of Ng and Rivera [10] for the detection of *Salmonella* spp., gradient PCR was used to determine the optimal annealing temperature for the effective amplification of the *STM4497* gene. Optimization of the PCR assay was tested using *S.* Typhimurium ATCC strain 14028 as the positive control, and gradient PCR was performed in triplicate and repeated three times for replicability and reproducibility. The PCR reaction components followed the manufacturer’s recommendations (Promega Corporation, WI, USA) and the protocol outlined by Ng and Rivera [10]. Details are provided in Table 2. Although amplification was observed at all tested temperatures in gel electrophoresis, the most effective results were achieved at 60 °C. This was evidenced by clear and intense bands at approximately 213 bp (Figure 1) for positive samples, with no bands observed for negative controls. The optimal PCR protocol for *STM4497* gene detection was found to be the following: initial denaturation at 95 °C for 2 min, then 30 cycles of denaturation at 95 °C for 30 s, annealing at 60 °C for 30 s, and extension at 72 °C for 2 min, with a final extension at 72 °C for 5 min using a thermocycler.

**Figure 2:**
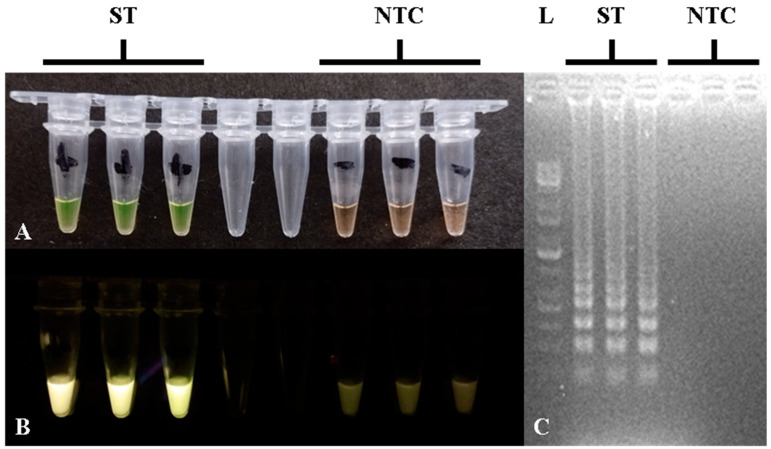
Closed-tube calcein-based LAMP assay of the *STM4497* gene showing positive and negative results in (**A**) color reaction; (**B**) fluorescence; (**C**) and gel electrophoresis. L—100 bp DNA molecular weight ladder; ST—*S.* Typhimurium; NTC—no-template control.

For the *STM4497* gene calcein-based closed-tube LAMP assay, the reaction components and protocols followed Balaga et al. [36], with some modifications, particularly for primers [32] and betaine concentration. The optimized LAMP assay component concentrations can be seen in Table 2. LAMP amplification was performed using a thermocycler at 65 °C for 60 min, followed by 80 °C for 2 min for the *Bst* enzyme inactivation. LAMP amplification in tubes showed yellow-green color or fluorescence using a blue LED light transilluminator, with gel electrophoresis showing ladder-like bands for positive results, while orange-brown color or absence of fluorescence and bands indicated negative results, as can be seen in Figure 2. The concentration of betaine was found to be critical for preventing non-specific amplifications, and the optimal concentration was at 1.5 M. Lower concentrations or absence of betaine led to false positive results through non-specific amplifications, which caused yellow-green reaction color and ladder-like bands in gel electrophoresis.

### 3.2. In Silico Analysis of STM4497 Gene PCR and Sequencing Confirmation

To determine the expected specificity and product size of the *STM4497* gene in PCR using Azinheiro et al.’s primers [32], F3 and B3 sequences were analyzed using in silico PCR against up-to-date prokaryotic genome sequences from the NCBI [33]. A total of 422 bacterial genomes, including 45 *Salmonella* spp. sequences (2 *S. bongori* strains, 1 *S. enterica* subsp. *arizonae* strain, 29 strains composed of 18 different *S. enterica* subsp. *enterica* non-Typhimurium serovars, and 13 strains of *S. enterica* subsp. *enterica* serovar Typhimurium), 65 *E. coli* strains, 13 *Enterococcus* spp. strains, 12 *Enterobacter* spp. strains, 12 *Klebsiella* spp. strains, 10 *Shigella* spp. strains, 57 *Pseudomonas* spp. strains, 43 *Listeria* spp. strains, 25 *Vibrio* spp. strains, 60 *Staphylococcus* spp. strains, and 80 *Bacillus* spp. strains, were subjected to the analysis. All 13 (100%) *S.* Typhimurium strains tested positive for the *STM4497* gene using F3/B3 primers with an expected amplicon size of 213 bp, while the 32 remaining non-Typhimurium *Salmonella* spp. strains and 377 non-*Salmonella* strains did not generate any product. This strongly suggests support for the suitability and specificity of the *STM4497* gene and the F3/B3 primer pairs for *S.* Typhimurium detection using PCR. To further confirm the *STM4497* gene, four PCR products—one from the positive control *S.* Typhimurium ATCC 14028 and three from positive samples (Marikina chicken, Manila pork, and San Juan beef)—were subjected to standard sequencing through ©Macrogen (Seoul, Korea) and sequence alignment and curation through Mega 11, which all resulted in 99.5–100% homology with the GenBank isolates of *Salmonella enterica* subsp. *enterica* serovar Typhimurium (accession numbers: CP149338.1, NZ_JAOTJO010000068.1) using BLAST-NCBI.

### 3.3. Specificity of the Optimized STM4497 Gene PCR and LAMP Assays

For the actual specificity testing of the *STM4497* gene using Azinheiro et al.’s primers [32], both the optimized PCR and LAMP assays were tested on a total of 18 (*n* = 18) ATCC bacterial strains composed of 9 non-*Salmonella* strains and 9 *Salmonella* strains in triplicate and repeated three times. Both the PCR and LAMP assays showed 100% specificity for the tested ATCC strains showing positive results, with PCR showing around 213 bp bands and LAMP showing yellow-green tubes and ladder-like bands for the two *S.* Typhimurium strains. Meanwhile, negative results, with PCR absence of bands and LAMP showing orange-brown tubes and no bands, were observed for the remaining 16 non-*S.* Typhimurium bacterial strains. The list of strains and their results for PCR and LAMP assays can be seen in Table 3.

### 3.4. Sensitivity of the Optimized STM4497 Gene PCR and LAMP Assays

**Figure 3:**
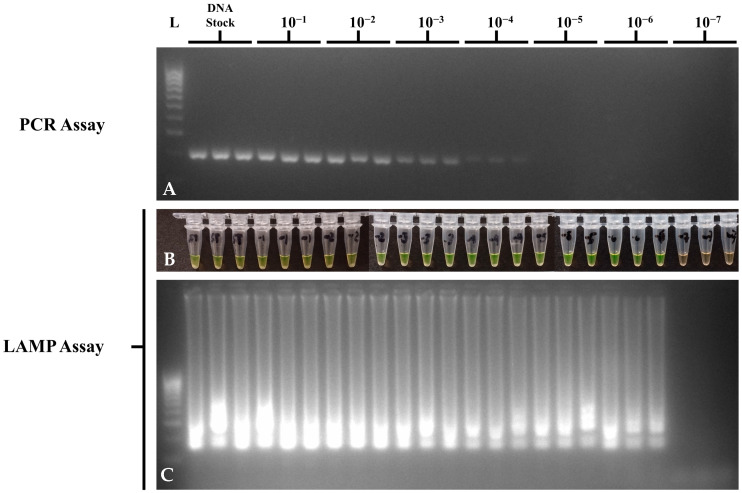
DNA dilution sensitivity of the optimized *STM4497* PCR and LAMP assays using *S.* Typhimurium ATCC 14028, showing (**A**) *invA* gene PCR gel electrophoresis; (**B**) *STM4497* gene LAMP tube; (**C**) and *STM4497* gene LAMP gel electrophoresis visualizations. L—100 bp DNA molecular weight ladder; ST—*S.* Typhimurium; NTC—no-template control.

Sensitivity assays for the optimized PCR and LAMP assays on *STM4497* gene detection were performed in triplicate and repeated three times using a dilution series of DNA and bacterial suspension of ATCC strain *S.* Typhimurium 14028 to determine the LOD in DNA at ng/μL and in bacteria at CFU/mL. For DNA dilution sensitivity, suspensions from stocks diluted to 10^−7^ were run in Multiskan SkyHigh Microplate Spectrophotometer (Thermo Fisher Scientific, Inc.) with 1X TE buffer as the reagent blank, in duplicate, to determine the DNA concentrations. The list of DNA concentrations and the results of PCR and LAMP assays per 10-fold DNA dilutions can be seen in Table 4. Concentrations below <1 ng/μL were calculated from the stock DNA extract due to the LOD of the MultiSkan SkyHigh Spectrophotometer (Thermo Fisher Scientific, MA, USA). Although both PCR and LAMP assays showed a LOD of <1 ng/μL, the optimized LAMP assay was able to perform detections at the 10^−6^ dilution or 220 fg/μL as compared to the PCR assay at the 10^−4^ dilution or 22 pg/μL (Figure 3). No-template controls showed no amplification in PCR and LAMP assays. This means that the LAMP assay was 100-fold more sensitive than PCR in detecting the *STM4497* gene in serially diluted DNA suspensions.

**Figure 4:**
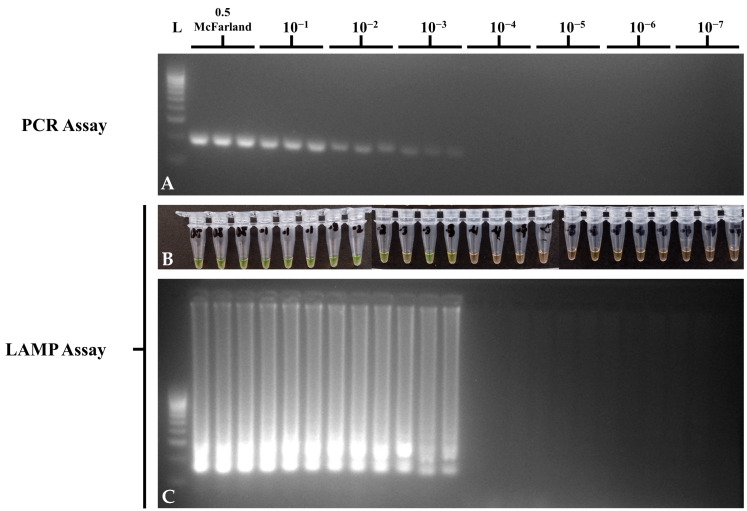
Bacterial culture dilution sensitivity of the optimized *STM4497* PCR and LAMP assays using *S.* Typhimurium ATCC 14028, showing (**A**) *invA* gene PCR gel electrophoresis; (**B**) *STM4497* gene LAMP tube; (**C**) and *STM4497* gene LAMP gel electrophoresis visualizations. L—100 bp DNA molecular weight ladder; ST—*S.* Typhimurium; NTC—no-template control.

For bacterial dilution sensitivity, *S.* Typhimurium ATCC 14028 suspensions from 0.5 McFarland to the 10^−7^ dilution were initially spread-plated in TSA to determine the colony counts for CFU/mL concentrations. The list of CFU/mL and the results of PCR and LAMP assays per 10-fold dilutions of bacterial suspensions can be seen in Table 5. Both the PCR and LAMP assays showed a LOD of 4.98 × 10^4^ CFU/mL (Figure 4), which suggests comparable sensitivity in detecting the *STM4497* gene in pure bacterial culture. No-template controls showed no amplification in PCR and LAMP assays.

### 3.5. Prevalence of Salmonella spp. in Retail Meat Samples

**Figure 5:**
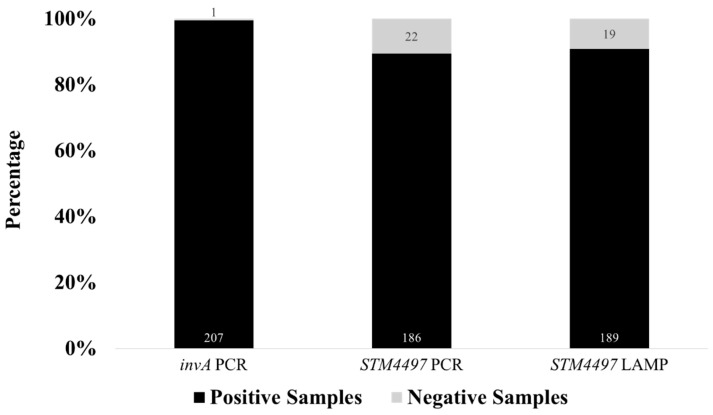
Detection rates of *invA* PCR for *Salmonella* spp. and *STM4497* PCR and *STM4497* LAMP for *S.* Typhimurium for 208 actual meat samples.

A total of 208 raw meat samples collected from wet markets around Metro Manila, Philippines, were processed from enrichment, selective enrichment, DNA extraction, and subsequently to *invA* gene PCR for *Salmonella* spp. detection before testing using the optimized *STM4497* PCR and LAMP assays for *S.* Typhimurium detection. For *Salmonella* spp. prevalence, 99.52% (207 out of 208) of the raw meat samples tested positive for the *invA* gene and were considered positive for *Salmonella* spp., which served as a basis for comparison for the detection of *S.* Typhimurium using the *STM4497* gene in PCR- and LAMP-based platforms (Figure 5). The single sample that tested negative for *Salmonella* spp. was obtained from beef collected in Manila City.

### 3.6. Performance of the Optimized PCR and LAMP Assays in Meat Samples

To evaluate the performance of the optimized closed-tube calcein-based LAMP assay, 208 DNA extracts from RV enrichments of raw meat samples were subjected to parallel testing with the optimized PCR assay for the detection of *S.* Typhimurium using the *STM4497* gene. While the *STM4497* gene PCR assay showed a positivity rate of 89.42% (186 out of 208) for *S.* Typhimurium, the LAMP assay showed a comparable detection rate of 90.87% (189 out of 208) (Figure 5). Both assays showed negative results for the one *invA* gene negative sample, which further suggests the specificity of *STM4497* to *Salmonella*—in particular, the Typhimurium serovar. A total of 16 samples were negative for both *STM4497* PCR and LAMP assays but positive for *invA* gene PCR, which suggests the presence of other non-Typhimurium *Salmonella* serovars. Meanwhile, a total of seven samples showed discordant results between *STM4497* PCR and LAMP assays, which suggests differences in the principle, sensitivity, tolerance, and potential for false results. Only two samples were positive for the *STM4497* PCR but negative for the LAMP assay, which suggests limitations of the optimized LAMP assay. On the other hand, a total of five samples were positive for the *STM4497* LAMP assay but negative for the PCR, which suggests higher sensitivity, tolerance, or possible false positives in the former assay.

Statistical analysis was performed using Fisher’s exact test to assess significant associations between two nominal variables (e.g., positive or negative results), with the null hypothesis stating that no significant association exists (Williams and Quave, 2019 [40]). Using SPSS version 26 (IBM, NY, USA), Fisher’s exact test showed a *p*-value of zero in a two-sided significance, which suggests the rejection of the null hypothesis and a strong statistical association between the *STM4497* gene PCR and LAMP assay results. This significant association suggests the corroboration of the *STM4497* gene PCR and LAMP assay results. Despite this, the LAMP assay required a shorter turnaround time to obtain the results. While the optimized PCR assay involved an amplification time of around 1 h and 30 min, including multiple temperatures and steps, the optimized LAMP assay only required 1 h of incubation at an isothermal temperature of 65 °C. In addition, the current study used a calcein-based visualization platform, which further decreases the time and workflow of LAMP, in contrast to PCR requiring gel electrophoresis, which can take up to 45 min longer.

## 4. Discussion

This study utilized the *STM4497* gene as a genetic marker for *S.* Typhimurium. Genes from *STM4488* to *STM4498* are known to encode *S.* Typhimurium-specific cytoplasmic proteins, such as type II restriction enzymes, helicases, ATPases, and proteases; however, some genes, including *STM4497*, remain putative and have unknown functions [41,42,43]. Nevertheless, several studies have used the *STM4497* gene after Kim et al. [44] showed its high specificity and superiority against more than 4400 genes obtained from the NCBI-BLAST database. This has also been explored on numerous samples, such as various foods, raw chicken meats from abattoirs and wet markets, poultry, raw shrimp, milk, and in diarrheic patients, using conventional, multiplex, and quantitative PCR platforms [45,46,47,48,49,50,51]. Other *S.* Typhimurium serovar-specific markers have also been explored. *fliC* and *fljB* genes encode for antigenic flagellar components, namely phase 1 and phase 2 flagellins [52,53]. However, both genes are often associated with other *Salmonella* serovars or even non-*Salmonella* strains, and they are also challenged with variations within serovars [45,54,55]. Meanwhile, the *typh* gene, originally discovered by Olsen et al. [56], has also been applied in PCR [57,58] and LAMP assays [4,32] to specifically detect *S.* Typhimurium. However, Azinheiro et al. [32] showed the superiority of the *STM4497* gene over the *typh* gene. The *mdh* gene encodes for a malate dehydrogenase enzyme that functions in the interconversion of malate and oxaloacetate for oxidative metabolic pathways [59]. It has often been used to detect monophasic variants of *S.* Typhimurium in PCR. However, *mdh* is also a known housekeeping gene, present in other *Salmonella* serovars and bacterial species [60,61], which presents homology from horizontal gene transfer and allelic polymorphisms, making it less serovar-specific. Meanwhile, other *STM* genes, such as *STM2755*, have shown comparable and high specificities with *STM4497* in PCR [54,62], albeit with limited serovars tested. Kim et al. [44] showed that some *STM* genes exhibited cross-reactivity against other *Salmonella* serovars, such as *STM2744* and *STM2755* with the Heidelberg serovar and *STM2630* and *STM2752* with other *S. enterica* subspecies.

LAMP assays can be implemented through numerous platforms that either require open-tube or closed-tube protocols. Open-tube LAMP assays for multiple procedures, such as the addition of *Bst* polymerase, DNA intercalating dyes, and loading of amplicons in gel electrophoresis, are known to have higher contamination risks. Some LAMP studies employ an initial denaturation step prior to the addition of the *Bst* polymerase to increase assay sensitivity [21,63]. However, LAMP can be designed as a rapid assay due to the strand displacement activity of the enzyme, which lessens amplification [19,64]. The current study did not implement a heat denaturation step and added the *Bst* polymerase prior to amplification. Visualization of LAMP assay products can be performed through different methods, such as turbidity from the precipitation of magnesium pyrophosphates during amplification, DNA intercalating dyes or gel electrophoresis, which require open-tube procedures that increase cross-contamination potential, and metal indicator dyes, which are applicable in closed-tube procedures. This study used calcein, a fluorescent metal indicator dye, which is often combined with a ratio of manganese and magnesium ions in LAMP assays. Prior to amplification, calcein molecules are quenched by manganese ions, which cause the solution to be orange-brown in color. However, when amplification occurs due to the presence of target genes, such as *STM4497*, manganese pyrophosphates are precipitated, and calcein then binds to the free magnesium ions, causing the reaction to appear yellow-green and fluorescent [35]. Meanwhile, HNB, another metal indicator dye, functions via the opposite mechanism by being initially quenched by magnesium ions, which become precipitated to free HNB [24]. However, one drawback is that excess concentrations of manganese ions are known to inhibit LAMP assays [65]. The current study utilized calcein and a generally low concentration of manganese of 1 mM, which remained highly sensitive and performed well against PCR. DNA intercalating dyes, such as SYBR Green I and EvaGreen, decreased amplification time with no reaction inhibition and better compatibility with *Bst* polymerase [24]. However, they can be toxic, more expensive, and they require open-tube protocols and visualization equipment. The current study used betaine to prevent non-specific amplifications and enhance the specificity of *STM4497* LAMP reactions. Betaine decreases the formation of secondary structures and nucleotide stacking, which often occur in GC-rich DNA templates [36,37]. The current study used an optimal 1.5 M betaine concentration, while lower concentrations or absence of betaine caused non-specific amplifications. Betaine remains unestablished among other studies. Whelan [66] reported that decreasing betaine concentration from 1 M to 0.5 M did not affect LAMP specificity in the detection of microRNAs, but its absence prevented any amplification. Meanwhile, LAMP assays developed by Foo et al. [67] and Balaga et al. [36] showed that lower betaine concentrations (<0.5 M) can lead to false positive results in the detection of *Entamoeba histolytica* and *Salmonella* spp., respectively. Like the current study, betaine at a concentration of 1.5 M or higher has also provided optimal LAMP amplifications in bovine embryo sexing [68], porcine circovirus genotyping [69], *Listeria monocytogenes* detection [70], and *Salmonella* spp. detection [71,72].

The F3 and B3 primers of LAMP assays are considered the most crucial primer sets, which can also serve as PCR primers. In LAMP assays, F3 and B3 primers facilitate strand displacement DNA synthesis, which frees FIP- and BIP-bound single-stranded DNA to allow the formation of loop structures at each end of the target gene, which then serve as templates for subsequent amplification cycles [73]. Analysis of the F3 and B3 primers has shown that the regions they target are in tandem and overlap with regions amplified by PCR primers [23,74,75]. Like this study, several studies have also applied LAMP outer primers to PCR assays for detection of various pathogens like *Toxoplasma gondii* [76], *Yersinia pestis* [77], *E. histolytica* [67], and *Salmonella* serovars [5,78,79,80]. Kalendar et al. [81] compared four different in silico PCR tools and showed that Bikandi et al.’s web-based tool [33] was faster than NCBI-BLAST, with comparable advantages. However, Bikandi et al. [33] used a smaller database, limited to the uploaded sequences, and mismatches can occur when shorter primers are used (10–12 nucleotides). Nonetheless, the *STM4497* F3 and B3 primers in the current study were 18 and 19 nucleotides in length, respectively.

Although the current study only tested a limited number of ATCC bacterial strains for actual specificity, the results reflect other *STM4497*-based PCR and LAMP studies. A multiplex PCR assay detecting *Salmonella* serovars Indiana, Enteritidis, and Typhimurium using serovar-specific genes *A7P63_0910*, *sdfI*, and *STM4497*, respectively, showed high specificity when tested against 348 *Salmonella* strains composed of 35 serovars and 12 non-*Salmonella* strains [82]. LAMP assay studies for the detection of *S.* Typhimurium and other pathogenic *Salmonella* serovars remain scarce [23]. However, different genes have been tested to detect *S.* Typhimurium in LAMP-based platforms, such as *typh*, *STM4495*, and *STM4497*. The *STM4497* primers used in the current study were previously implemented in quantitative LAMP or commercial LAMP platforms and have also shown 100% specificity against more *Salmonella* serovars, such as Liverpool and Wentworth, and other non-*Salmonella* bacterial species like *Citrobacter*, *Listeria*, and *Campylobacter* [6,32]. Meanwhile, another study relying on turbidimetry used a different primer set for the *STM4497* gene [83]. Recently, Gong et al. [84] developed a one-step LAMP assay combined with CRISPR Cas12b using the *STM4497* gene, which showed 100% specificity against 30 reference strains. Pavan Kumar et al. [4] used the *typh* gene and showed 100% specificity against a limited number of serovars, but the gene was later found to cross-react with other *Salmonella* serovars, namely Oranienburg, Wentworth, and another non-Typhimurium *Salmonella* strain [32]. Chen et al. [5] used *STM4495* for LAMP-based *S.* Typhimurium detection and showed 100% specificity but only tested limited strains. Meanwhile, the *JYM79_16920* gene showed cross-reactions against six *Salmonella* serovars: Abony, Adjame, Thompson, Bredeney, Give, and Quebec [85]. Therefore, the specificity of the optimized LAMP assay in this study is well supported and suggests the suitability of *STM4497* as a *S.* Typhimurium-specific marker.

The current closed-tube and calcein-based LAMP assay possessed higher sensitivity over PCR in detecting *S.* Typhimurium DNA (ng/μL) while corroborating its sensitivity in pure bacterial culture (CFU/mL). The LAMP-based assay possessed a LOD of 220 fg/μL, which was 100-fold more sensitive than the PCR assay, which possessed a LOD of 22 pg/μL. Previous studies have also shown the superiority of LAMP assays over PCR-based platforms. However, LAMP assay sensitivity can be affected by numerous factors, such as the target gene and primers used, the type of visualization or amplification method, amplification duration, reaction component concentrations, and the presence of inhibitors from reagents or sample matrices. A SYBR Green I-based LAMP study targeting the *typh* gene also showed 100-fold higher sensitivity, detecting up to 2 pg DNA in LAMP compared to only 200 pg in PCR [4]. Meanwhile, a Midori Green-based quantitative LAMP assay with DMSO, and using the current study’s primers, showed a 10-fold higher sensitivity over qPCR, detecting up to 160 pg/μL compared to only 1.6 ng/μL, respectively [6]. The addition of loop primers can also be beneficial in decreasing the reaction time and increasing LAMP assay sensitivity and specificity [64]. Azinheiro et al. [32] showed higher sensitivity with a LOD of 0.00438 ng/µL using Garrido-Maestu et al.’s primers [6] for *STM4497* with the addition of Loop F. The current study showed higher sensitivity than the previous two studies despite using the same primer sets, which suggests the potential advantage of using this gene, Loop F, and a calcein-based LAMP platform. Similarly, a comparable sensitivity of 250 fg, using the same primers for *STM4497* in the current study, was observed using a commercial real-time and closed-tube LAMP platform [86]. Although the addition of loop primers and reaction time have been associated with sensitivity in LAMP assays [32,78], increasing amplification duration may not always be beneficial [87]; hence, this study used a typical reaction time of 60 min [23,36]. The 10^4^ CFU/mL LOD in the current study was also reflected in previous studies detecting *Salmonella* spp. in pure bacterial culture using calcein-based LAMP colorimetry and fluorescence [88,89] and real-time LAMP [90,91] platforms. Interestingly, Ravan and Amandadi [83] optimized a highly sensitive *STM4497*-based LAMP assay using a different primer set to the current study, with a LOD of only 10 colonies/reaction tube in pure culture, 10^3^ CFU/mL without pre-enrichment, and 10 CFU/mL with pre-enrichment of only four hours, using simple turbidimetric methods and gel electrophoresis. The number of primers also affected sensitivity in a calcein-based LAMP study detecting a novel gene (*gene62181533*) targeting *Salmonella* spp. [92]. This study showed that while four primers (B3, FIP, BIP, and LF) detected 4.1 CFU/mL, combinations of five or six primers all showed the same higher sensitivity of 4.1 CFU/mL. These results suggest that the presence of complete outer primers and either one or two loop primers provides higher sensitivity and is more optimal for LAMP assays.

LAMP visualization platforms and reaction components have also been shown to contribute to assay sensitivity. A study comparing calcein, eriochrome black, and HNB showed that while the former two detected up to 0.001 ng of DNA, HNB showed a higher sensitivity of up to 0.0001 ng of DNA [65]. Similarly, Goto et al. [93] revealed that calcein was ten times less sensitive than HNB and SYBR Green I, attributed to the manganese inhibition principle. However, Fischbach et al. [24] demonstrated that both HNB- and calcein-based LAMP assays possessed equal sensitivities, with calcein having the advantage of increased contrasts due to fluorescence under UV light. Combining calcein and HNB has also been explored in a novel LAMP assay to detect foodborne pathogens, which resulted in easier visualization and result discrimination without the need for fluorescence [25].

The prevalence of *Salmonella* spp. in the current study (>90%) is higher than in previous studies in Metro Manila, Philippines (40–60%), with different rates depending on the sample types (raw or processed), location types (wet markets or abattoirs), or establishment accreditation [10,11,12,38]. However, these studies implemented complete conventional culture until isolation, followed by PCR. The current study attempted to reduce the workflow and turnaround time by eliminating plating and isolation through DNA extraction directly from selective enrichment. While isolation of *Salmonella* may be advantageous in phenotype and genotype characterization, it can underestimate *Salmonella* numbers and delay turnaround times, especially for extensive biochemical and serovar identification. Numerous studies have shown the advantages of *Salmonella* detection in the enrichment steps, such as higher detection, sensitivity, and decreased turnaround time. A study comparing the performance of PCR in the detection of *Salmonella* spp. and its serovars showed that RV-PCR detected 128% more positive samples for *Salmonella* spp. than complete conventional culture and with a turnaround time of only two days instead of the typical seven days [52]. Similarly, a comparison with non-selective enrichment still showed the superiority of RV-PCR over conjunction with complete conventional culture and non-selective enrichment for genus level detection [94]. Serovar detection in these two studies showed similar sensitivity in RV-PCR and complete conventional culture–PCR, with the *fliC* gene being used to detect *S.* Typhimurium. Goodman et al. [95] also showed a 100-fold increase in the LOD for *Salmonella* spp. including the Dublin serovar, where a commercial qPCR system and an automated DNA extraction system were coupled with RV selective enrichment, compared to culture in selective agars. Another study showed that sensitivity was also affected by the type of selective enrichment broth and extraction procedure used, wherein RV-PCR was only superior over selenite-cystine broth and Müller-Kauffmann tetrathionate when the phenol-chloroform DNA extraction procedure was used and not boiling or salting out methods [96]. Like the current study, a 95% *Salmonella* spp. detection rate was obtained from *invA* gene PCR after enrichment with Gram-negative broth as compared to 60% in culture methods alone [31]. However, there is also the possibility of cross-reactions or cross-contaminations that lead to false positive results in some LAMP studies due to detection at the enrichment steps rather than isolation [97].

A potential cause of the discordant results between PCR and LAMP assays, particularly due to lack of LAMP amplification, is that conventional LAMP assays are often unable to tolerate mismatches between primers and templates. Inner primers (FIP and BIP, with 40 nucleotides) often have twice as many nucleotides as outer primers (F3 and B3, with 18–22 nucleotides), which presents more possibility for mismatches with the DNA template [98] than PCR. This can be addressed using a high-fidelity *Bst* DNA polymerase [99]—using additives like Tween 20, DMSO, or tetramethylammonium chloride [100]—or through improved primer design [101]. Manganese and cadmium ions have also been shown to increase the mutation rates in LAMP assays through binding with dNTPs and causing template-independent synthesis [102]. Conversely, discordant results—where samples test positive in LAMP but negative in PCR—may indicate the higher sensitivity of the LAMP assay or the possibility of false positive results. Numerous studies have shown the benefits of LAMP over PCR following enrichment steps. Enrichment experiments in spiked stool and blood samples showed that while the LAMP assay, using four primers and gel electrophoresis, was able to detect *Salmonella* serovar Typhi after ten hours of incubation, qPCR required at least two hours more incubation [103]. LAMP assays are also more tolerant of inhibitors. They can tolerate hematin found in oxidized blood, humic acid found in agriculture, antibodies, and bile salts, as well as culture media components like salts and amino acids [104,105]. Ge et al. [106] reported a multi-laboratory validation study comparing the FDA-BAM standard method with the enrichment-LAMP assay and showed that both methods were comparable, and LAMP efficiency for *Salmonella* spp. detection was not affected by the culture media used. Another LAMP-based *Salmonella* detection study using the *hisJ* gene showed that higher detection limits were obtained through addition of the RV enrichment step than direct BPW detection, which suggests possible inhibitory components from sample matrices [107]. Several reports have also shown that selective enrichment media for *Salmonella*, such as RV and tetrathionate broths, are inhibitory to PCR assays [108,109]. LAMP assays can also cause non-specific and non-template reactions from primer–primer binding and carry-over contaminations, causing false positives due to more and longer primers [110,111]. The current study utilized betaine to decrease this phenomenon; however, other methods to reduce non-specific amplifications include additives like DMSO, pullulan, and graphene oxide, enzymes like UDG and DNAzyme, or the implementation of confirmatory tests, such as the CRISPR/Cas system, lateral flow assay, and machine learning [111,112]. In another study, the use of five primers instead of six also showed reduced false positive rates or misamplifications in the detection of SARS-CoV-2 using qLAMP [113].

There are some limitations to the current study, such as the need for in-depth analysis of LAMP primer amplification efficiency, exploration and comparison of more primer sets, evaluation of internal amplification controls, higher number of actual strains in specificity testing, implementation of actual fitness-for-purpose calculations, which involve meat spiking for the determination of positive and negative predictive values, or detailed cost analysis comparisons between the methods. The current study provides crucial data for the performance of a closed-tube and calcein-based LAMP assay for the detection of *S.* Typhimurium against a more standard method, such as PCR. Further studies are recommended to enhance the current method. These may include optimizing pre-LAMP processes, shortening the enrichment steps or developing direct detection protocols, and improving primer design. Exploration of the reaction additives, more objective and accurate visualization platforms, and investigation of LAMP inhibitors or factors contributing to false results are also needed. Additionally, expanding the detection to other *Salmonella* serovars and foodborne pathogens, conducting more field tests using various sample matrices, and comparing the results with culture and serological standards are strongly encouraged. Nevertheless, the developed and optimized closed-tube and calcein-based LAMP assay coupled with enrichment steps is a potentially powerful alternative or complementary tool to PCR for the detection of *S.* Typhimurium in raw meat samples.

## 5. Conclusions

In the Philippines, where *Salmonella* contamination remains high throughout the food animal industry, there is a pressing need for alternative or complementary tools to detect relevant *Salmonella* serovars, such as Typhimurium. The developed closed-tube and calcein-based LAMP assay targeting the *STM4497* gene showed 100% specificity and 100-fold higher sensitivity when compared directly with PCR. This presents potential grounds for a simpler, faster, and more powerful surveillance method for *S.* Typhimurium detection that lowers the turnaround time from the traditional seven days—up to serological identification—to just three days, with a shorter amplification time than PCR. This study also optimized a PCR assay for *S.* Typhimurium detection using the same outer primers for *STM4497*, which was tested in silico and confirmed through DNA sequencing. Both the LAMP and PCR assays showed high detection rates (>89%) for *S.* Typhimurium in retail meat samples from Metro Manila after selective enrichment steps. The high prevalence of *S.* Typhimurium suggests potential underestimations of *Salmonella* contamination and presents a possible food safety and consumer health risk. The findings of this study may contribute not only to strengthening surveillance capacity but also to informing policy development and guiding mitigation strategies in agricultural practices, food animal processing, and retail, ultimately supporting the safety of producers, consumers, and the broader public. However, the findings in the current study are not without limitations, and further validations of the method and strengthening of assay repeatability and reproducibility are necessary. Hence, although the current *STM4497*-based closed-tube and calcein-based LAMP assay is potentially a powerful alternative or complementary detection tool for *S.* Typhimurium, further research is recommended.

## Figures and Tables

**Table 1:** Primer sequences and references for PCR and LAMP assays.

Target Gene	Primer	Sequence (5′-3′)	Reference
***Salmonella* spp. PCR Primers**			
*invA*	*invA* ^F^	ACAGTGCTCGTTTACGACCTGAAT	[31]
	*invA* ^R^	AGACGACTGGTACTGATCTAT	
***Salmonella enterica* serovar Typhimurium LAMP Primers**			
*STM4497*	*STM* ^FIP^	ACCTGCAGCTCATTCTGAGCAGTCAAAAACAACGGCTCCGG	[32]
	*STM* ^BIP^	GAAAAGGACCACAAGTTCGCGCTCAGTGAGCATGTCGACGAT	
	*STM* ^F3^	AGCCGCATTAGCGAAGAG	
	*STM* ^B3^	GCGGTCAAATAACCCACGT	
	*STM* ^LoopF^	TCAAAAATCCAGAACCCAATCTCA	

**Table 2:** Reaction components and concentrations of the optimized *STM4497* gene PCR and LAMP assays for the detection of *S.* Typhimurium.

**PCR ASSAY (*STM4497* Gene)**		
**Components**	**Concentration Tested**	**Optimized**
GoTaq^®^G2	1x	1x
F3	0.4 µM	0.4 µM
B3	0.4 µM	0.4 µM
Nuclease-Free H_2_O	Make up 12.5 µL	Make up 12.5 µL
DNA Template	1 µL	1 µL
**LAMP ASSAY (*STM4497* Gene)**		
**Components**	**Concentration Tested**	**Optimized**
Calcein	64 µM	64 µM
MnCl_2_	1 mM	1 mM
MgSO_4_ (Added)	3 mM	3 mM
Isothermal Amplification Buffer	1x	1x
dNTPs	1.4 mM	1.4 mM
FIP/BIP Primers	1.6 µM	1.6 µM
F3/B3 Primers	0.2 µM	0.2 µM
Loop F Primers	0.4 µM	0.4 µM
Nuclease-Free H_2_O	Make up 25 µL reaction	Make up 25 µL reaction
Betaine	1–1.5 M	1.5 M
*Bst* Polymerase 2.0	320 U/mL	320 U/mL
DNA Template	1 µL	1 µL

**Table 3:** Specificity testing of the optimized *STM4497* gene PCR and LAMP assays for ATCC bacterial strains (*n* = 18).

ATCC Strain	PCR	LAMP
**Non-*Salmonella* strains (*n* = 9)**		
*Acinetobacter baumanii* BAA 1605	−	−
*Escherichia coli* 25922	−	−
*Escherichia coli* O157:H7 43888	−	−
*Escherichia coli* O78:H11 35401	−	−
*Enterococcus faecalis* 14506	−	−
*Klebsiella pneumoniae* 13883	−	−
*Klebsiella pneumoniae* 700603	−	−
*Pseudomonas aeruginosa* 10145	−	−
*Vibrio parahaemolyticus* 13204	−	−
***Salmonella enterica* subsp. *enterica* serovar strains (*n* = 9)**		
Anatum 9270	−	−
Choleraesuis 7001	−	−
Choleraesuis 10708	−	−
Enteritidis 13076	−	−
Enteritidis 49223	−	−
Heidelberg 8386	−	−
Newport 6962	−	−
Typhimurium 14028	+	+
Typhimurium 25241	+	+

**Table 4:** DNA dilution sensitivity showing DNA concentrations in ng/μL per 10-fold dilutions of *S.* Typhimurium ATCC 14028 DNA and results for the optimized *STM4497* gene PCR and LAMP assays.

10-Fold Dilution	Concentration	PCR	LAMP
Stock	220 ng/μL	+	+
1	28.2 ng/μL	+	+
2	2.59 ng/μL	+	+
3	* 220 pg/μL	+	+
4	* 22 pg/μL	+	+
5	* 2.2 pg/μL	−	+
6	* 220 fg/μL	−	+
7	* 22 fg/μL	−	−
NTC	−	−	−

* Concentrations <1 ng/μL were calculated from stock solutions to circumvent the limitation of the plate reader; NTC—no-template control.

**Table 5:** Bacterial culture dilution sensitivity showing CFU/mL per 10-fold dilutions of *S.* Typhimurium ATCC 14028 culture and results for the optimized *STM4497* gene PCR and LAMP assays.

10-Fold Dilution	LOD (CFU/mL)	PCR	LAMP
0.5 McFarland	4.98 × 10^7^	+	+
1	4.98 × 10^6^	+	+
2	4.98 × 10^5^	+	+
3	4.98 × 10^4^	+	+
4	4.98 × 10^3^	−	−
5	4.98 × 10^2^	−	−
6	49.8	−	−
7	4.98	−	−
NTC	−	−	−

NTC—no-template control.

## Data Availability

The original contributions presented in this study are included in the article. Further inquiries can be directed to the corresponding author.
